# The Cancer-Immune Set Point in Oesophageal Cancer

**DOI:** 10.3389/fonc.2020.00891

**Published:** 2020-06-04

**Authors:** Robert Power, Maeve A. Lowery, John V. Reynolds, Margaret R. Dunne

**Affiliations:** ^1^Department of Surgery, Trinity College Dublin, Dublin, Ireland; ^2^Trinity St. James Cancer Institute, Trinity College Dublin, Dublin, Ireland

**Keywords:** cancer immunology, immunotherapy, oesophageal cancer, immune checkpoint inhibitors, prognostic markers

## Abstract

Immunotherapy has achieved long-term disease control in a proportion of cancer patients, but determinants of clinical benefit remain unclear. A greater understanding of antitumor immunity on an individual basis is needed to facilitate a precision oncology approach. A conceptual framework called the “cancer-immune set point” has been proposed to describe the equilibrium between factors that promote or suppress anticancer immunity and can serve as a basis to understand the variability in clinical response to immune checkpoint blockade. Oesophageal cancer has a high mutational burden, develops from pre-existing chronic inflammatory lesions and is therefore anticipated to be sensitive to immune checkpoint inhibition. However, both tumour- and patient-specific factors including the immune microenvironment, the microbiome, obesity, and host genetics contribute to an immune set point that confers a lower-than-expected response to checkpoint blockade. Immunotherapy is therefore currently confined to latter lines of treatment of advanced disease, with no reliable predictive biomarker of response. In this review, we examine oesophageal cancer in the context of the cancer-immune set point, discuss factors that contribute to response to immunotherapeutic intervention, and propose areas requiring further investigation to improve treatment response.

## Introduction

Oesophageal cancer is the sixth most common cause of cancer-associated mortality worldwide and represents a major global health challenge (1). Oesophageal cancer is divided into squamous cell carcinoma (OSCC) and adenocarcinoma (OAC). The incidence of OAC has increased markedly in the western world within the last 40 years and is thought to arise from a multi-step inflammatory dysplastic transformation from the precursor lesion of Barrett's oesophagus (BO). Stomach acid and bile reflux and visceral obesity predispose individuals to both BO and OAC (2, 3). In contrast, OSCC accounts for 90% of oesophageal cancer worldwide and tobacco or alcohol consumption are the main risk factors (4, 5). As 5-year survival rates are <20% for these cancers (6) and systemic therapy confers a response in only a minority of patients, alternative treatment options are urgently needed (7, 8).

**Figure 1:**
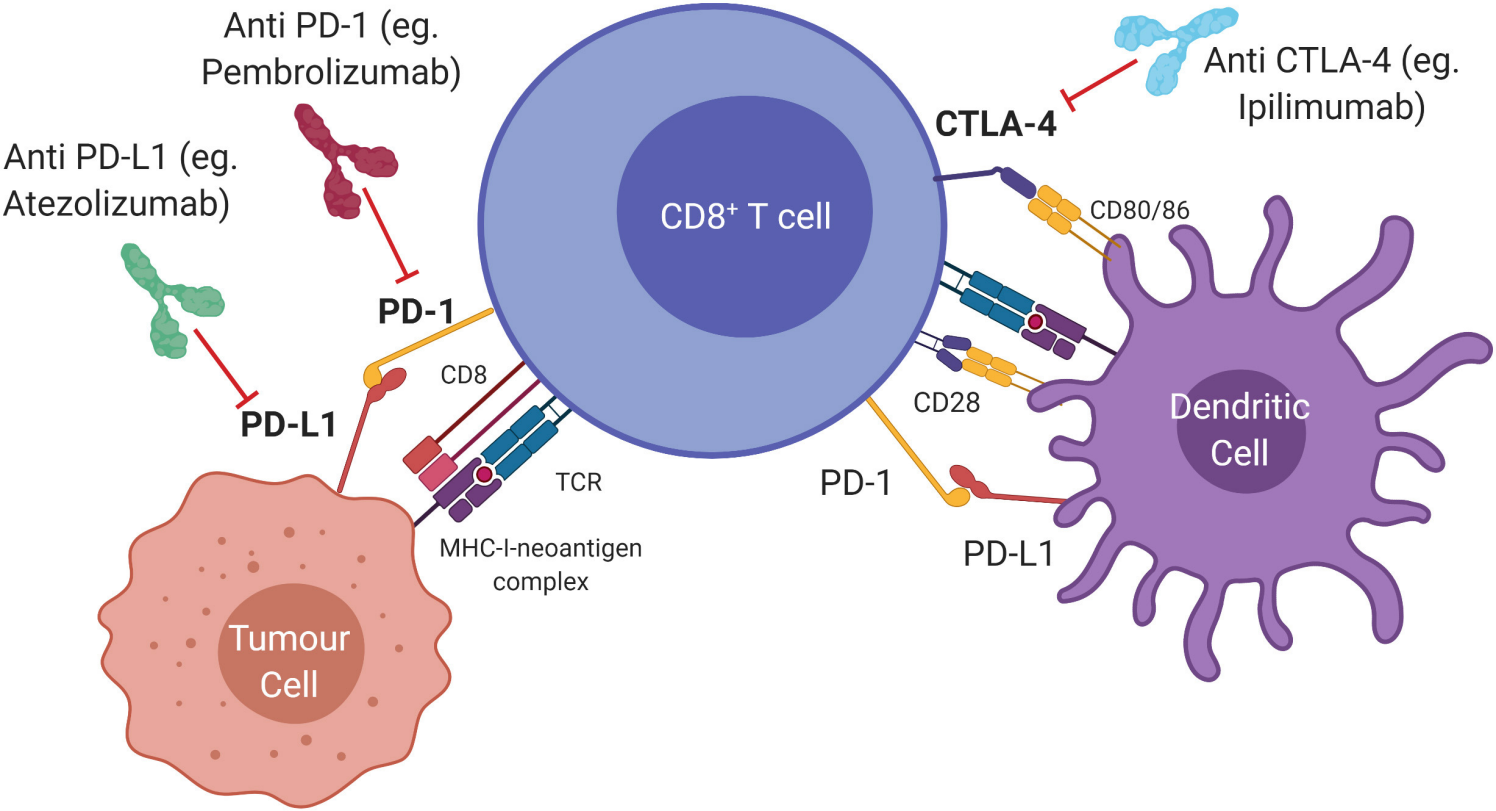
Immune checkpoints and therapeutic targets in the anti-tumour immune response. Cytotoxic T lymphocyte antigen 4 (CTLA4) is a ligand expressed by T cells which prevents T cell activation and can be blocked by Ipilimumab (anti CTLA4). Activated T cells express programmed cell death protein 1 (PD1) which transmits an inhibitory signal that attenuates cytolytic activity when bound to programmed death ligand 1 (PDL1). Monoclonal antibodies that interfere with the PD1/PDL1 interaction (anti PDL1; anti PD1) allow re-invigoration of T cells.

Several regulatory pathways, so-called “immune checkpoints” involved in immune homeostasis are hijacked by cancer cells as a means of evading the host immune response (Figure 1). The first to be targeted was cytotoxic T lymphocyte antigen 4 (CTLA4), expressed constitutively by regulatory T (T_reg_) cells, and by activated T cells. CTLA4 inhibits T cell activation by binding to costimulatory molecules CD80/CD86 on antigen-presenting cells or tumour cells (9). Inhibition of this pathway by antibody ligation, also known as immune checkpoint inhibition (ICI), has led to major clinical advances in the treatment of advanced melanoma (10). Programmed cell death-ligand 1 (PDL1, encoded by *CD274*) and 2 (PDL2, encoded by *PDCD1LG2*) are expressed by antigen presenting cells and some tumours, and bind to programmed cell death protein 1 (PD1, encoded by *PDCD1*) on effector T cells (11). This generates an inhibitory signal, resulting in attenuated cytotoxic activity. Administration of a monoclonal antibody that blocks the PDL1/PD1 interaction allows reinvigoration of inactivated T cells (12). This approach has led to durable clinical responses in melanoma, non-small-cell lung cancer (NSCLC), head and neck squamous cell carcinoma (HNSCC), renal cell carcinoma and urothelial carcinoma (13–16). Combination approaches incorporating both PD1/PDL1 and CTLA4 blockade, have seen clinical approval in mismatch-repair deficient colorectal cancer, renal cell carcinoma, and hepatocellular carcinoma (17–19).

The rationale to utilise immunotherapy for oesophageal cancer treatment stems from a recognised link with precursor chronic inflammatory lesions and a high mutational burden, suggesting an activated immune response which could be exploited for therapeutic benefit (20). However, as will be discussed in this review, the impact of immunotherapy on patient outcomes in oesophageal cancer to date has been limited (21). An improved understanding of the immune landscape of oesophageal cancer is therefore urgently required to develop effective immunotherapeutic strategies and to select patients likely to benefit from treatment. To conceptualise the myriad of factors that determine a favourable clinical response, a “cancer-immune set point” has been proposed; reflecting the equilibrium between factors that promote or suppress anticancer immunity and a threshold that must be overcome to generate an effective immune response to a tumour (22). A patient with a low set point responds to immunotherapy easily, while the converse is true in patients with a high set point. The immune set point of an individual is determined by tumour specific factors such as tumour genome, precursor lesions and the tumour microenvironment (TME), alongside the external factors of obesity, host genetics, viral infection, and the human microbiome. This review aims to evaluate what is known about each of these factors in the setting of oesophageal cancer, in order to better understand ways in which immunotherapeutic strategies can be improved.

## THE Cancer-Immune Set Point

### The Tumour Genome

The overall mutational burden of a tumour increases the probability that some mutations are immunogenic and can be presented as neoepitopes on major histocompatibility class I (MHC-I) molecules. This stimulates a CD8^+^ T cell response and favourably affects the immune set point. This can be assessed clinically by measuring tumour mutational burden (TMB), defined as the number of asynchronous mutations per mega-base pair (mut/Mbp) which has been correlated to response to immune checkpoint inhibition (ICI) in a variety of tumour types, including oesophageal and gastric cancer (23). Relative to other malignancies, OAC has a relatively high mutational burden at 9.9 mut/Mbp, which is ranked 5th of 30 tumour types in terms of mutational burden, malignant melanoma, and NSCLC being the first and second, respectively (24, 25). The Cancer Genome Atlas (TCGA) found that chromosomal instability was a cardinal genomic feature of OAC and shared with gastric cancer (26). Whole genome sequencing of 129 OAC samples, as part of the International Cancer Genome Consortium (ICGC), established 3 subgroups based on mutational signatures. The “mutagenic” subgroup displayed the highest TMB, neoantigen burden, and CD8^+^ tumour infiltrating lymphocyte (TIL) density which may lead to an increased response to ICI (27). More recently, a combined multi-omic characterisation of 551 OAC samples has revealed a three-way association between hypermutation, activation of the *Wnt* pathway (associated with T cell exclusion from tumour parenchyma) and loss of immune signalling genes such as *B2M* (β2 microglobulin, a component of MHC-I) (28, 29). Hypermutation is associated with higher immune activity, while *Wnt* dysregulation and loss of *B2M* is associated with immune escape (30). This provides an acquired mechanism through which OAC may prevent immune surveillance induced by a high mutational burden, potentially offering an explanation for the observed lack of response to checkpoint inhibition.

Specific genomic alterations may also influence the immune set point, independent of overall mutational burden. Amplifications of receptor tyrosine kinases are frequent events in OAC, accounting for 32% of cases which display amplification of *ERBB2* (encoding the HER2 receptor) (26). HER2-positive breast cancer is associated with a distinctive immune landscape (31). Like breast cancer, HER2-positive OAC can be targeted by trastuzumab which could potentially modify the immune set point by antibody-dependant cellular cytotoxicity (32). Adding trastuzumab to standard chemotherapy in patients with metastatic gastroesophageal adenocarcinomas with HER2 overexpression showed a higher objective response rate and a significant increase in overall survival (33). However, tumour heterogeneity has been proposed as a barrier to success of HER2 targeted treatments in the gastroesophageal setting, unlike breast cancer (34). Other common driver mutations, including *TP53* and *KRAS* can promote PD-L1 expression, immune evasion, and immunosuppressive remodelling of the microenvironment in mouse models of pancreatic cancer (35, 36). In a study of resected OAC samples *KRAS* amplifications were a poor prognostic marker (37). Interestingly, amplifications in *PIK3CA*, present in just 5% of cases, correlated with a T cell rich inflammatory microenvironment and were associated with increased survival. There is a need to further characterise the genomic correlates of immune cell infiltration in oesophageal cancer, as has been carried out in colorectal cancer (38), to fully evaluate the impact of these driver mutations on the immune set point.

The genomic landscape of OSCC is distinct from OAC with upregulation of the *Wnt, SOX2*, and *TP63* pathways. The latter two genes are required for squamous epithelial differentiation which may explain a similar mutation signature to head and neck SCC (26, 39). OSCC also has a lower mutational burden than OAC; one cohort (*n* = 62) of tumours displayed a mean TMB of 3.9 mut/Mbp (40). In a direct comparison between the two subtypes, 3% of OSCC tumours were TMB-high (>17 mutations/Mbp) compared to 8% of OAC. However, a higher proportion of these same OSCC samples expressed PDL1 (41 vs. 9%) which suggests that the higher TMB of OAC does not necessarily correspond to increased PDL1 expression (41). In summary, the two subtypes of oesophageal cancer are genomically distinct, and this differential mutational burden contributes to divergent immune set points.

### The Immune Landscape of Precursor Lesions

**Figure 2:**
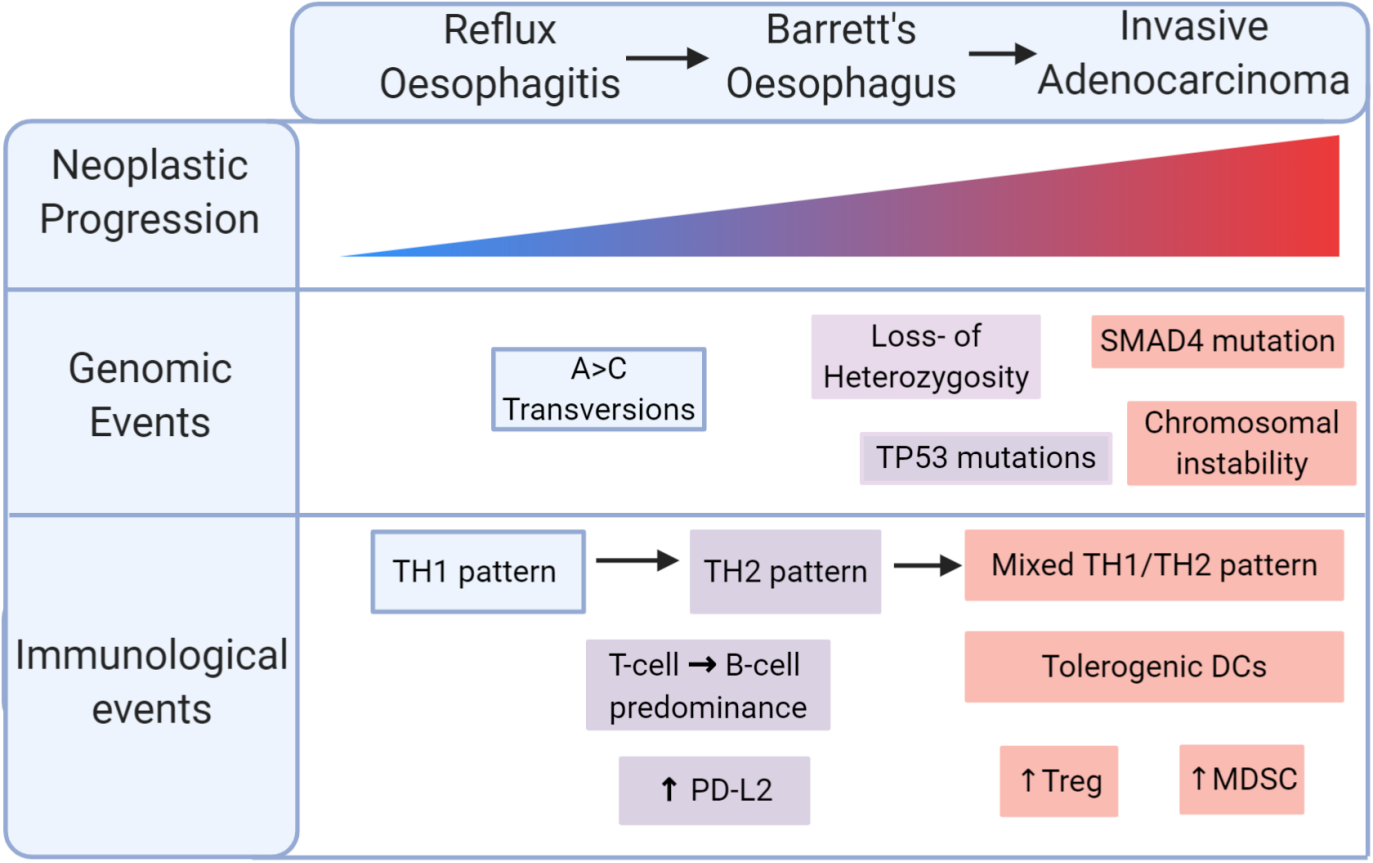
Immunological progression in the malignant transformation to oesophageal adenocarcinoma (OAC). Reflux oesophagitis is accompanied by a TH_1_ pattern of inflammation which shifts to a TH_2_ pattern in Barrett's oesophagus. Malignant transformation is marked by a mixed TH_1_/TH_2_ pattern with tolerogenic dendritic cells (DCs), regulatory T (Treg) cells, myeloid derived suppressor cells (MDSCs).

Despite differences in genetic drivers of disease, both types of oesophageal cancer share a background in chronic tumourigenic inflammation. OAC in particular is an exemplar model of inflammation-driven cancer, arising from a background of BO metaplasia, driven by chronic reflux, and characterised by intense inflammatory immune cell infiltration, summarised in Figure 2. Cytokine profiling and more recent T cell immunophenotypic studies have associated reflux oesophagitis with a predominantly T helper type 1 (TH_1_) type cytokine profile, predominated by IFN-γ and interleukin 2 (IL2) expression, whereas BO displays a humoral-type TH_2_ profile, associated with immunosuppression (42–45). Supporting this, a recent single-cell flow analysis found a shift from T cell to B cell predominance as normal tissue progresses to BO specialised intestinal metaplasia (46). This TH_2_ polarisation drives upregulation of epithelial PDL2 in models of BO and OAC, suggesting that cytokine profile can indirectly induce T cell exhaustion (47). During this malignant progression, dendritic cells are rendered tolerogenic, promoting T_reg_ cell formation, and tumour progression (48). At the end of this sequence, OAC is associated with a mixed TH_1_ and TH_2_ profile, impaired T cell trafficking, and reduced levels of effector T cells (Figure 2) (49). Together, these data indicate that inflammation is a key initiator of the metaplasia-dysplasia-carcinoma sequence, but an immunosuppressive phenotype, potentially an adaptive response to inflammatory stress, enables transformation to OAC.

### An Immunosuppressive Tumour Microenvironment

**Figure 3:**
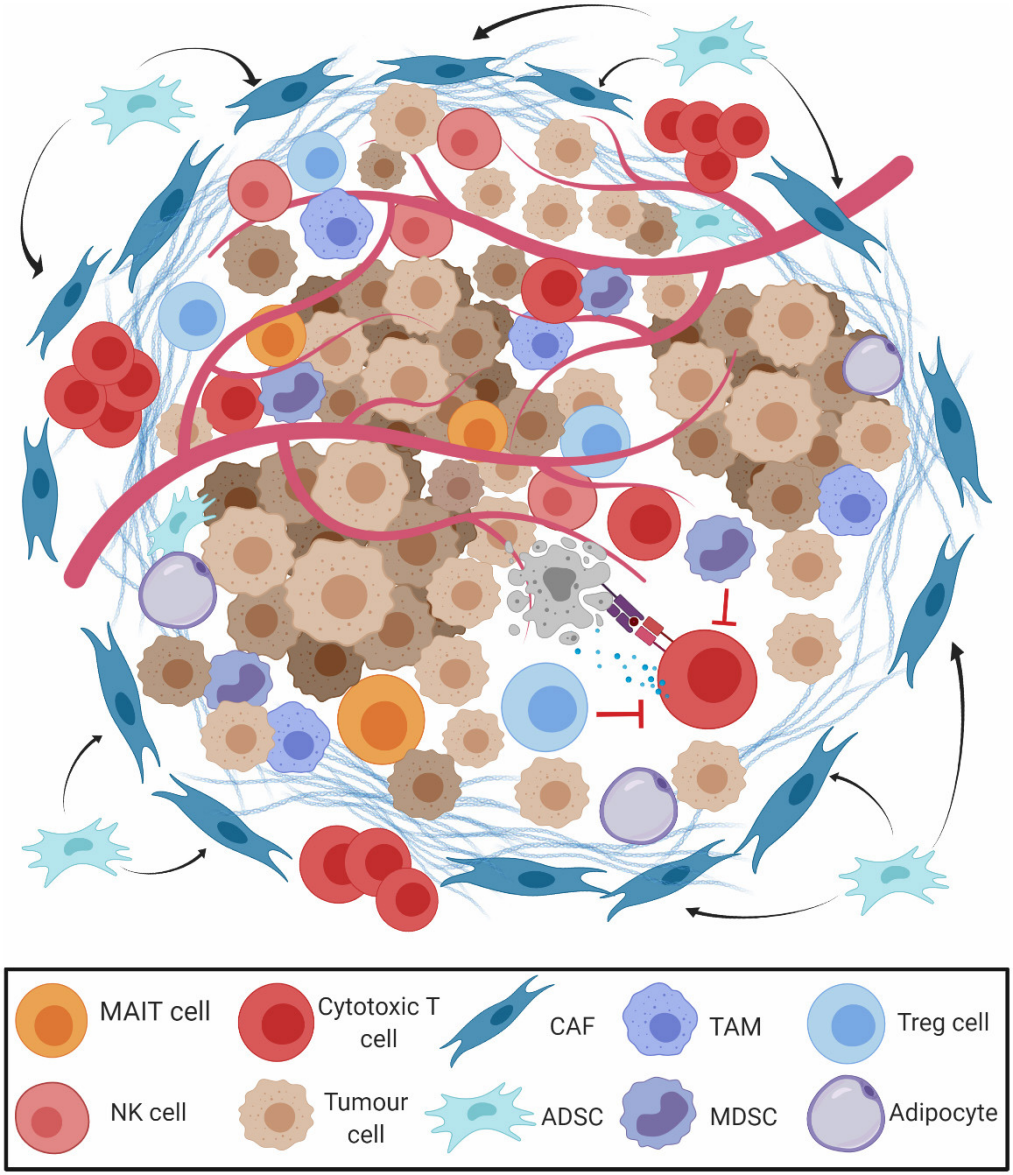
The tumour microenvironment (TME) in oesophageal adenocarcinoma. The presence of M2-polarised tumour-associated-macrophages (TAM), regulatory T cells (Treg), and myeloid derived suppressor cells (MDSCs) restrict the action of cytotoxic CD8^+^ T cells (CD8 T cells), natural killer (NK) cells, and mucosa associated invariant T (MAIT) cells. Cancer associated fibroblasts (CAFs) and adipocytes derived stem cells (ADSCs) secrete extracellular matrix (ECM) and prevent migration of effector T cells to the tumour parenchyma.

The mass of cells surrounding cancerous cells is often reprogrammed to induce a pro-tumorigenic milieu, known as the tumour microenvironment (TME) (Figure 3) (50, 51). Some elements of the immune environment can promote anticancer immunity, including conventional CD8^+^ cytotoxic and CD4^+^ helper T cells, and unconventional lymphocyte subsets with potent tumour-killing ability, such as natural killer (NK) cells (52), gamma-delta (γδ) T cells (53), and mucosa associated invariant T (MAIT) cells (54). Tumours exhibiting high levels of lymphocytic infiltration are referred to as “hot” tumours, those without “cold,” and tumours with intermediate or ineffective infiltration are referred to as “altered” (55). CD8^+^ TILs are observed in OAC tissue microarrays, and high levels at the tumour centre have been reported to be positive prognostic indicators (56–58). CD4 helper T cells, although not prognostic alone, have been recently shown to play an essential role in assisting CD8 T cell anti-tumour responses in many cancer types (59). Interestingly, elevated expression of the CD4 T cell antigen presentation molecule, HLA-DR, was noted to be an independent favourable prognostic indicator in OAC (60) and other gastrointestinal tumour types, further highlighting the importance of CD4 T cells involvement in antitumour responses. A large molecular profiling study on 18,000 tumours across 39 malignancies including oesophageal cancer showed that γδ T cells and a MAIT cell associated gene *KLRB1* ranked as the most favourable markers of overall survival (61), highlighting a more important role for unconventional lymphocytes as mediators of antitumor immunity than previously thought. Lymphocyte activation state was also shown to affect immune cell prognostic ability. MAIT cells comprise a portion of CD8^+^ TILs in OAC tumours and display a diminished effector capacity (62). NK cells are also potent antitumor effectors, but intra-tumoral NK cells display markers of exhaustion in OAC. These cytotoxic cells may be abundant in the immunogenic environment of ICGC-mutagenic OAC (27), suggesting an intact immune response that could be potentiated by PD1 blockade, or potentially by other novel means of therapeutic targeting.

Other constituents of the TME promote a pro-tumour milieu. Cancer-associated fibroblasts secrete extracellular matrix proteins and chemokines, excluding CD8^+^ T cells from the tumour parenchyma (63). The vast majority (93%) of OAC tumours contain cancer-associated fibroblasts which interfere with T cell receptor signalling and leukocyte trafficking, conferring a poor prognosis (64). While “classically” activated M_1_-macrophages have antitumor qualities, “alternatively” polarised M_2_-macrophages produce immunosuppressive growth factors and cytokines that drive progression from BO to OAC (65, 66). Myeloid-derived suppressor cells (MDSCs, defined by CD11b^+^Gr1^+^ coexpression), and FoxP3^+^ T_reg_ cells restrict antitumor CD8^+^ T cell cytotoxicity and are recruited by TH_2_ cytokines in the tolerogenic milieu of OAC (67, 68). T_reg_ cell abundance in resected OAC samples is linked with advanced stage and poor response to treatment (69–71). Populations of these tolerogenic cells may be prominent in the non-mutagenic ICGC subsets of OAC and contribute to a non-T cell inflamed immune profile.

In OSCC, there is an abundance of effector T cells and NK cells adjacent to cancer cells (72). Around 40% of OSCC tumours display high (>10%) levels of TILs, suggesting an intermediate level of immune infiltration. Similar to OAC, levels of CD8^+^ TILs are a favourable prognostic factor in OSCC (73) but a large subset are confined to the stroma (74). Interestingly, high levels of stromal CD8^+^ TILs are a stronger prognostic factor than intratumoural TILs in both early and late stage OSCC, suggesting that effector function is not limited by their location. The presence of M_2_-polarised tumour associated macrophages is associated with angiogenesis, PDL1 expression, and poor prognosis in resected OSCC samples (75, 76). Like OAC, populations of MDSCs and CAFs restrict CD8^+^ T cell function in OSCC and may reduce efficacy of PD1 blockade (64, 77). Infiltrating FoxP3^+^ T_reg_ cells are also seen in OSCC but are not an independent predictor of survival. Levels of FoxP3 TILs solely correlates with effector CD8/4^+^ levels, implying a less potent suppressive role in OSCC. Tumour cell PDL1 expression (>1%; the percentage of viable tumour cells that stain for PDL1 by immunohistochemistry) in OSCC is around 48%, compared to 23% in OAC (78, 79), potentially contributing to T cell exhaustion in the TME. The intermediate TIL infiltration, presence of suppressive cell populations, and immune checkpoint expression is typical of an altered-immunosuppressed tumour profile; suggesting different components of the TME shape the immune landscape of OAC and OSCC.

This distinction between hot, altered, and cold tumours is useful but overly simplifies the complex cancer-immune equilibrium to solely a T cell mediated response. Like many biological characteristics, the immune contexture of oesophageal cancer exists on a patient-specific continuum, and a broader view of anticancer immunity is therefore required. For example, high expression of B cells follicular helper T cell (T_FH_) markers correlate with survival in colorectal cancer (80). T_FH_ cells secrete CXCL13 which supports organisation of B cells into compartments known as tertiary lymphoid structures (TLS) (81). “Mature” TLS can promote anti-tumour immunity through antibody dependant cellular cytotoxicity and antigen presentation (82, 83) while “immature” TLS may suppress T cell dependant immunity by expressing IL10 and PDL1 (84). Presence of mature TLS in tumours can predict response to immunotherapy in melanoma, sarcoma, and renal cell carcinoma (85–87). More recently, type 2 innate lymphoid cells (ILC2s) have emerged as tissue specific enhancers of anti-cancer immunity and amplify the efficacy of PD1 blockade in pancreatic cancer (88). Evaluating the role of these emerging elements of anti-tumour immunity in oesophageal cancer could describe a more nuanced picture, expanding the immune microenvironment beyond the dichotomy of “hot” and “cold.”

### The Gut and Tumour Microbiome

There is growing evidence that the diversity and content of the human microbiome is a component of an individual's inherent immune profile. Preclinical studies have long suggested that the response to anti-PD1/PDL1 therapy is contingent on an intact gut microbiome, and this is supported by recent research in melanoma, NSCLC and colorectal cancer patients (89, 90). In these studies, patients that responded to ICI had increased microbial diversity, increased microbial anabolic activity, high levels of *Faecalibacterium* and low levels of *Bacteroidales* in their gut microbiome. Increased CD8^+^ TILs, higher levels of circulating effector T cells and a preserved cytokine response to PD1 blockade were found in patients with a putative favourable microbiome, suggesting that the gut microbiome influences antitumor immunity (91, 92). The gut microbiota can stimulate chemokine production in human colorectal tumours to influence TIL recruitment, shifting the immune set point (93). Furthermore, 11 low-abundance strains of human commensal bacteria were found to induce interferon-γ producing CD8^+^ T cells in the intestine, and colonisation enhances efficacy of ICI in mouse models of colorectal cancer (94). In addition to the gut microbiome, the tumour microbiome has also been found to impact the immune setpoint in pancreatic cancer (95). Long term survivors had higher tumour microbiome diversity which shaped a favourable immune microenvironment, with augmented recruitment and activation of T cells.

Of interest, the eradication of *Helicobacter pylori* has been epidemiologically associated with an increase in OAC development, as has gastroesophageal reflux disease (GERD), and both conditions may alter the distal oesophageal microbiome (96–98). Indeed, oesophageal microbial diversity is altered in progression from BO to OAC (99). Microbiome phenotyping of OAC patients revealed a high abundance of *Fusobacterium nucleatum*, relative to normal oesophageal tissue (100, 101). These tumour samples were associated with a high degree of immune infiltration, and upregulation of MHC class II on intratumoral antigen-presenting cells following anti-PD1 therapy (100). In tandem, antibiotic use is associated with a lack of response to PD1 blockade in OSCC along with other cancers, which has been hypothesised to be mediated by intestinal dysbiosis (102).

In NSCLC and melanoma, faecal microbiota transplant (FMT) from human ICI responders improved response to ICI in mice, raising a possibility of a microbiome based therapeutic intervention (91, 92). A pilot study that subjected three ICI-refractory melanoma patients to FMT from ICI-responders has reported preliminary results (103). FMT increased intratumoural CD8^+^ TILs in recipients, and this translated into a clinical and radiological response in two of three patients. A similar trial is currently ongoing in oesophageal cancer (NCT04130763). There is a need to further understand the immunomodulatory role of the microbiome in non-T-cell inflamed tumours such as oesophageal cancer, since there may be potential here to discover novel treatment targets or adjuvants, which may ultimately predict and improve clinical response to ICI.

### Obesity

Obesity has a multifaceted effect on the immune system and is beginning to be appreciated as a determinant of the cancer-immune set point (104). Excess adiposity drives a state of chronic low-level inflammation, leading to increases in the number of adipose tissue-derived stem cells, fibroblasts, and extracellular matrix in the TME (105). Adipose tissue-derived stem cells exert an immunomodulatory role through suppression of NK cell, B cell, and cytokine responses (106) and contribute to interstitial fibrosis (107, 108). In preclinical models of obesity associated cancers, obesity increases levels of MDSCs, M2-polarised macrophages and tolerogenic dendritic cells in the TME (109, 110). Given the strong relationship between obesity and OAC development, OAC is uniquely poised as a model for understanding the interplay between obesity and anticancer immunity (111, 112). In obese OAC patients, effector T cells are found to preferentially migrate to the omentum and the liver rather than infiltrating OAC tumours (113, 114). This is mediated by the CX3CL1 chemokine and may contribute to the non-T-cell inflamed immune profile of OAC (115).

The role of obesity in the cancer-immune set point has clinical implications. The protective effect of mild obesity (30–34.9 kg/m^2^) has also been noted in certain cancers, termed the “Obesity Paradox” (116), where obesity is associated with prolonged survival in melanoma and NSCLC patients treated with immunotherapy (117). This mechanism has been proposed to involve leptin signalling, which drives T cell exhaustion, increases PD1 expression and impairs effector capacity. This attenuates antitumor immunity and promotes tumour progression but concurrently increases sensitivity to PD1 blockade (118). This is paradoxical, as an impaired immune response would be expected to decrease the efficacy of immunotherapy. Obesity associated immune alterations also provide targets for therapy; M2 polarisation of macrophages can be prevented by specific inhibitors and apoptosis of obesity associated MDSC populations in the TME can be induced by liver X receptor-β (LXRβ) agonists (110, 119). A combinatorial approach to immunotherapy may be useful in obesity associated cancers, including OAC.

### Host Genetics

Genetic variation in immune response genes has been hypothesised to contribute to the inherent immune profile of a tumour and the immune set point of a cancer patient (22). An expression quantitative trait loci (eQTL) analysis found that common germline genetic variants can influence immune gene expression in 24 cancer types. Oesophageal cancer was not part of this dataset. Expression of *ERAP2* (endoplasmic reticulum aminopeptidase 2), a pan-cancer gene associated with MHC-I antigen processing, predicted survival in bladder cancer patients receiving ICI therapy (120). A total of 103 germline gene signature QTLs were associated with immune cell abundance in the TME. This highlights that germline genetics are an underappreciated determinant of immune gene expression and immune cell infiltration, potentially providing a new means of stratifying patients for ICI treatment. Patient HLA genotype, particularly heterozygosity of HLA-I alleles (*HLA-A, HLA-B, HLA-C*) is associated with more efficient neoantigen presentation, and extended survival in melanoma patients treated with ICI (121). More recently, HLA evolutionarily divergence as measured by sequence divergence between HLA-I alleles was found to predict ICI response in NSCLC and melanoma (122). No studies have assessed HLA genotype in ICI outcomes in oesophageal cancer. Germline loss-of-function in the *TLR4* gene has been associated with lack of response to chemo- and radiotherapy in breast cancer patients, putatively due to an effect on T cell antigen priming (123). A similar effect has been described in the *P2RX7* purinergic receptor, which activates the *NLRP3* inflammasome to produce IL1β, essential in CD8^+^ T cell priming (124). Immunogenic cell death involves release of ATP and HMGB1 which bind to *TLR4* and *P2RX7*, respectively, to promote tumour antigen presentation. However, in both subtypes of oesophageal cancer, loss-of-function in *TLR4* was unexpectedly associated with improved cancer-specific survival (71). Loss-of-function mutations in *P2XR7* were not associated with a survival difference but were associated with intratumoral T_reg_ cell infiltration (71). Most research has focused on the tumour as a genomic predictor of response to ICI while the host genome has been left relatively unexplored. Future work should further elucidate the effect of germline genetic variation on the cancer-immune set point in oesophageal cancer, as there is evidence that oesophageal cancer may have unique traits which may prove useful in predicting ICI responses.

### Viral Infection

Tumours secondary to viral infection, such as Epstein Barr Virus (EBV), or Human Papilloma Virus (HPV) can also express neoantigens derived from viral open reading frames (125, 126). The “EBV associated” gastric cancer subset has increased PDL1 expression, immune cell signalling*, PIK3CA* mutations, and reliable response to ICI (127). HPV-associated oropharyngeal cancer is associated with increased PDL1 expression and durable responses to immunotherapy (15, 128). OAC may also be associated with EBV in 0–6% of cases (129–131), and although this link is less robust than with gastric cancer, EBV tumour testing may represent a potential predictive biomarker to ICI (132). HPV has also been associated with OSCC in numerous case studies, especially in Asian populations (131, 133) but this association may reflect the worldwide prevalence of HPV rather than a causal relationship (26, 134). Although specific viral antigens have not yet been identified as common predictive markers in either subtype of oesophageal cancer, direct administration of viral antigens has shown potential in boosting general anti-tumour immunity (135). In a recent study, intratumoral injection of an unadjuvanted influenza vaccine reduced growth in preclinical models of melanoma and NSCLC and augmented PD1 blockade. Vaccination increased levels of tumour antigen-specific CD8^+^ T cells and dendritic cells in the TME, effectively converting a tumour from immunologically “cold” to “hot.” Data from 300 patients with lung cancer showed that those who received influenza vaccination had a longer overall survival time (136). This strategy presents a cost-effective way to potentially shift the immune set point and transform oesophageal cancer to a T cell inflamed phenotype. However, further study is required since it is also observed that vaccination may increase risk for adverse immune events in cancer patients receiving ICI therapy (137).

### Wider Environmental Factors

Immunity in humans can also be influenced by wider environmental exposures including drug intake, sun exposure, diet, and smoking. Chronic statin therapy, for example, is associated with altered response to the influenza vaccine in older people (138). Decreased exposure to sunlight is associated with increased serum levels of IL6 and C-reactive protein (139). This may be linked to vitamin D metabolism, as the *VDR* (vitamin D receptor) has differential seasonal expression (139). Vitamin D-*VDR* activation suppresses Wnt signalling and promotes anti-tumour immunity in melanoma (140), and expression of an enzyme that degrades vitamin D (*CYP24*) is a poor prognostic marker in OSCC (141), suggesting that vitamin D may be a link between diet, sun exposure and immunity. The incidence of both NSCLC and OSCC is associated with tobacco consumption and the carcinogenic effects of smoking confers a unique mutational signature (24). This signature is associated with response to PD1 blockade in NSCLC (142). In OSCC, however, smoking status was not associated with TIL frequency or PDL1 expression (143), suggesting a less robust relationship between smoking and anti-cancer immunity.

The molecular pathological epidemiology (MPE) framework can help integrate these complex dietary, lifestyle, environmental, and microbiome factors with multi-omic data to create a complete picture of the immune set point in oesophageal cancer (144). Such an approach has associated high levels of plasma 25-hydroxyl vitamin D with a lower risk of colorectal cancer with an intense T cell infiltrate (145). MPE approaches can also integrate microbiome data with immune phenotypes; *Fusobacterium Nucleatum* colonisation is associated with less immune infiltration in human colorectal tumours and may impair NK cell cytotoxicity (146, 147). This MPE framework can be used to evaluate the relationship between microbiome, environmental factors and immunity in oesophageal cancer, which can further aid understanding of an individual's immune set point.

## Immunotherapy Trials in Oesophageal Cancer

Multiple clinical trials have evaluated PD1/PDL1 blockade, both alone and in combination in patients with OAC (Table 1). Tumour expression of PDL1, as determined by the combined positive score (CPS; the number of PDL1 staining cells divided by the total number of viable tumour cells, multiplied by 100) has been used to select and stratify patients on ICI trials (157). Early trials have established the safety of the anti-PD1 agents pembrolizumab and nivolumab in the chemorefractory setting. The phase 1/2 CHECKMATE-032 study investigated the role of nivolumab and/or ipilimumab in oesophageal and gastric cancer and included 26 patients with OAC (149). It found an objective response rate (ORR) of 24% in patients treated with nivolumab and ipilimumab, and this was 31% in patients with PDL1 positive (>1%) tumours. The ATTRACTION-2, phase III study, found that nivolumab improved overall survival (OS; 5.2 vs. 4.1 months, *p* < 0.0001) in heavily pretreated gastric (GC) or gastroesophageal junction cancer (GEJC). A limitation of this trial was that it only enrolled Asian patients, which have been shown to have a different tumour immune signatures, and better outcomes in GEJC clinical trials compared to non-Asian patients (158). In the KEYNOTE-059 phase II study of pembrolizumab in previously treated GC or GEJC, the ORR was 11.6%, with a longer median duration of response in PDL1^+^ patients (16.3 vs. 6.9 months) (150). Based on these results, the FDA granted approval of pembrolizumab in recurrent GC or GEJC that overexpresses PDL1. In the phase 3 KEYNOTE-181 trial, pembrolizumab as second-line therapy for advanced oesophageal cancer (OAC/OSCC) did not improve OS in the whole population, compared to chemotherapy, but did improve survival for patients with strong expression of PDL1 (CPS ≥10) (156).

**Table 1:** Completed clinical trials of immunotherapy in oesophageal cancer.

**Study**	**Phase**	**Disease setting**	**Prior lines**	**Intervention**	**Results**
Doi et al. (148) (KEYNOTE 028)	Ib	Advanced OAC (*n* = 27) and OSCC (*n* = 65)	≥2	Pembrolizumab	ORR = 24/83 (30%)
Janjigian et al. (149) (CheckMate−032)	I/II	Advanced OAC (*n =* 59), GEJC (*n =* 75) and GC (*n =* 19)	≥2	Nivolumab + Ipilimumab vs. Nivolumab	ORR = 24 vs. 12% Median OS = 6.9 vs. 4.8 mo
Fuchs et al. (150) (KEYNOTE-059)	II	GEJC (*n* = 133) or GC (*n* = 126)	≥2	Pembrolizumab	ORR = 11.6% in PD-L1^+^ patients, 15.5% in PD-L1^−^ patients
Shitara et al. (151) (KEYNOTE-061)	III	Advanced GEJC (*n* = 89) or GC (*n* = 207)	1	Pembrolizumab vs. Paclitaxel	Median OS 9.1 vs. 8.3 mo (HR: 0.82; *p* = 0.0421)
Shah et al. (152) (KEYNOTE-180)	II	Advanced OAC (*n* = 58) and OSCC (*n* = 63)	≥2	Pembrolizumab vs. Placebo	ORR = 12/21 (9.9%)
Janjigian et al. (153) (NCT0295453)	II	HER2+ advanced gastroesophageal adenocarcinoma (*n* = 24)	None	pembrolizumab, trastuzumab plus chemotherapy	ORR = 20/24 (83%) Median PFS = 11.4 mo
Kudo et al. (154)	II	Advanced OSCC (*n =* 64)	1	Nivolumab	ORR = 11/64 (17%)
Kang et al. (155) (ATTRACTION-2)	III	Advanced GEJC or GC	≥2	Nivolumab vs. placebo	Median OS 5.3 vs. 4.14 mo (HR = 0.63, *p* < 0.0001)
Kato et al. (78) (ATTRACTION-3)	III	Advanced OSCC (*n =* 419)	1	Nivolumab vs. Investigator's choice of chemotherapy	Median OS 10.9 vs. 8.4 mo (HR: 0.77 *p* = 0.019)
Kojima et. al. (156) (KEYNOTE-181)	III	Advanced OAC (*n* = 227) and OSCC (*n =* 401)	1	Pembrolizumab vs. Investigators choice of chemotherapy	Median OS 9.3 vs. 6.7 mo (HR: 0.69, *p* = 0.0074) No difference in ITT group

*OAC, oesophageal adenocarcinoma; OSCC, oesophageal squamous cell carcinoma; GEJC, gastroesophageal junction carcinoma; ORR, objective response rate; OS, overall survival; PFS, Progression-free survival; HR, hazard ratio; ITT, intention-to-treat; mo, month*.

The phase Ib KEYNOTE-028 study evaluated the safety of pembrolizumab in PDL1 positive oesophageal cancer, the majority (65/92; 78%) of which were OSCC (148). The ORR was 30% and response was correlated to an interferon-γ gene expression signature. In KEYNOTE-181, a trend was observed favouring responses in patients with OSCC (156). This, along with the results of KEYNOTE-180 led to the FDA approval of pembrolizumab in metastatic OSCC with a CPS ≥10 after ≥1 line of therapy. Nivolumab was also evaluated in chemorefractory OSCC in a phase II trial, showing a modest ORR (17%) but manageable toxicity (154). More recently, the ATTRACTION-3 phase III study investigated the use of nivolumab in the second line treatment of advanced OSCC (78). Patients in the Nivolumab arm had a prolonged OS (10.9 vs. 8.4 months, *p* = 0.019), and less toxicity compared to chemotherapy regardless of PDL1 status. However, most (96%) patients were of Asian ethnicity, potentially limiting applicability to wider patient populations.

### Future Combination Approaches

Combining immunotherapy with chemotherapy, radiotherapy or targeted therapy is currently being investigated to boost the modest response rate of oesophageal cancer to ICI. The precise delivery of radiotherapy and the resulting induction of immunogenic cell death may convert a tumour into an *in-situ* vaccine through the release of damage-associated molecular patterns (DAMPs) (157). Calreticulin, ATP and HMGB1 are all DAMPs released by radiation-induced cell death that promote efficient neoantigen processing by antigen presenting cells and priming of CD8^+^ T cells (159). DNA released following radiation-induced cell damage can stimulate the cGAS-STING pathway, triggering type I interferon production (160, 161). Finally, radiotherapy can upregulate pre-existing neoantigen expression, and remodel the cellular composition of the TME (162). These effects enhance tumour immunogenicity and form the preclinical rationale of ongoing trials of ICI and chemoradiotherapy in resectable oesophageal cancer (NCT02735239).

There is also evidence that trastuzumab, a HER2 targeted therapy can have a synergistic effect with ICI. A phase II trial of 1st line pembrolizumab alongside trastuzumab and chemotherapy in HER2^+^ OAC and GC found an encouraging ORR of 87% (153). This may be related to induction of immunogenic cell death by trastuzumab, releasing neoantigens, and stimulating a specific CD8^+^ T cell response (163). This prompted the opening of the larger phase III KEYNOTE-811 trial (NCT03615326) which is currently recruiting patients. Cytotoxic chemotherapy can have genotoxic effects and general novel tumour neoantigens. Other cytotoxic agents (anthracyclines, cyclophosphamide, oxaliplatin, and taxanes) induce immunogenic cell death, increasing tumour adjuvanticity (164). This type of ICI combination is being investigated in the phase III KEYNOTE-590 study of pembrolizumab alongside 5-fluorouracil and cisplatin in the first line treatment of locally advanced/metastatic OAC and OSCC (165, 166).

## Concluding Remarks

In spite of many preclinical and clinical studies, immunotherapy in oesophageal cancer currently remains confined to 2nd or 3rd line treatment of metastatic disease, with no unequivocal predictive biomarker available. These modest results are likely due to a high cancer-immune set point, where ICI is not sufficient to drive progression of the cancer immunity cycle. This is despite a high mutational burden in OAC, and an intermediate level of CD8^+^ TILs in OSCC and OAC, suggesting an altered-immunosuppressed immune profile; where antitumor cytotoxicity is limited by soluble inhibitory mediators and suppressive cell populations in the TME (159). Less well-characterised aspects of the cancer immune set point in including obesity in OAC, and the microbiome in both subtypes, should be further explored as potential determinants of this immunosuppressive phenotype.

Although our knowledge of the individual components of the cancer-immune set point in oesophageal cancer has grown, the macroscopic picture is still poorly understood. We propose a systems biology approach integrating multi-omic tumour profiling with individual patient data to accurately predict antitumor immune responses. Optimally such an approach combines tumour genomics, immunohistochemistry, and peripheral blood assays to generate a “Cancer Immunogram” and integrate complex immune biomarkers (167). This paradigm has been applied in NSCLC, where whole-exome sequencing and RNA-seq separated 20 patients into personalised Immunograms (168), a proof-of-concept that such an approach may be clinically feasible. However, integrating these genome and immune based biomarkers with environmental exposures is needed to fully account for interpatient variability in immunotherapy response. In this sense, the MPE framework may prove vital in evaluating the role of obesity, the microbiome and other external determinants of the immune set point in oesophageal tumours.

Conceptualising the cancer-immune set point provides clinicians and researchers with a crucial framework connecting the innumerate factors that determine response to immunotherapy. The immune landscape of oesophageal cancer is heterogeneous and is contingent on both patient- and tumour-specific variables. We anticipate that successful immuno-oncology drug development in oesophageal cancer will be dependent on leveraging knowledge of these factors to develop personalised treatment strategies, involving a combination of ICI and radiation or systemic therapy to elicit a T cell inflamed phenotype.

## Author Contributions

RP and MD wrote the first draft of this paper. All authors contributed to editing and preparation of the final draft.

## Conflict of Interest

ML reports a Consulting/Advisory Role for Agios, Celgene, and Roche/Genentech. The remaining authors declare that the research was conducted in the absence of any commercial or financial relationships that could be construed as a potential conflict of interest.
